# The Lymph Self-Antigen Repertoire

**DOI:** 10.3389/fimmu.2013.00424

**Published:** 2013-12-16

**Authors:** Cristina C. Clement, Laura Santambrogio

**Affiliations:** ^1^Department of Pathology, Albert Einstein College of Medicine, New York, NY, USA; ^2^Department of Microbiology and Immunology, Albert Einstein College of Medicine, New York, NY, USA

**Keywords:** lymph, antigen processing, antigen presentation, MHC class II

## Abstract

The lymphatic fluid originates from the interstitial fluid which bathes every parenchymal organ and reflects the “omic” composition of the tissue from which it originates in its physiological or pathological signature. Several recent proteomic analyses have mapped the proteome-degradome and peptidome of this immunologically relevant fluid pointing to the lymph as an important source of tissue-derived self-antigens. A vast array of lymph-circulating peptides have been mapped deriving from a variety of processing pathways including caspases, cathepsins, MMPs, ADAMs, kallikreins, calpains, and granzymes, among others. These self peptides can be directly loaded on circulatory dendritic cells and expand the self-antigenic repertoire available for central and peripheral tolerance.

## Lymph Formation

The lymphatic fluid originates from the interstitial fluid which bathes every parenchymal organ and it is generated through a process of ultrafiltration of the plasma, circulating through the blood capillaries, as well as by the addition of metabolic and catabolic products collected from the tissue of origin (1–4).

Once the proteins have been filtered into the extracellular space, they will not re-enter the blood circulatory system by uptake into the venous capillaries as previously thought. Indeed, what was known as the Starling principle has been recently revisited and it is now apparent that almost all the extravasated fluid will be drained into the lymphatics (5).

In addition to the proteins and molecules originating from plasma ultrafiltration, the interstitial fluid will be further enriched with products derived from tissue/organ catabolism/metabolism (6–13).

The interstitial fluid will then drain into open end lymphatic capillaries and hence forth be called lymph (14, 15). The pre-nodal lymph will flow into progressively larger collectors up to the draining lymph nodes (500–600 in humans), disseminated throughout the body. All lymph passes through one or more lymph nodes and each node collects lymph from a distinct region of the body (4). Thus, a molecular signature of tissue-specific self proteins is collected in each node.

## Lymph Proteome, Degradome, and Peptidome

During the last two decades there has been an increasing interest in the analysis of the protein composition of human and rodent lymph under physiological and pathological conditions and in the comparative analysis with plasma samples (6–13, 16–20). This analysis has been elusive for many years due to the difficulty in cannulating lymphatics, which run much deeper then veins and have a smaller diameter and more fragile walls. Additionally, mass spectrometric techniques employed just a few years ago were much less sensitive than they are now in mapping proteins expressed at low levels within scarce amounts of collected fluid.

Proteomic profiles have been reported for human, rodent, bovine, and ovine lymph, and two major conclusions can be drawn from the compilation of these: (6–13, 16–20).

(i) The lymph proteomic profile is not merely overlapping with the one from the plasma but qualitative and quantitative differences can be appreciated; indeed the lymph proteome appears to be enriched in products deriving from tissue and cellular metabolism/catabolism, organ remodeling, extracellular matrix processing, and cellular apoptosis.
(ii) The proteomic molecular signature reflects the tissue from where the lymph is collected and the organ’s physiological or pathological condition. Indeed tissue-specific proteins have been mapped in the lymph collected from capillaries draining specific organs and infectious or inflammatory tissue conditions are reflected in proteomic changes in lymph more so than in plasma.

The proteomic profile of the lymph also revealed the presence of several low molecular weight products composed by fragments, derived from protein processing, and short peptides (12). A similar degradome and peptidome was previously mapped in the plasma and serum and other biological fluids; the most comprehensive analysis so far reports up to 6000 peptides, identified with high confidence in mouse serum (21). Several more groups reported on the low molecular weight cleaved proteome and peptidome revealing the remarkable richness of protein fragments and naturally processed peptides present in lymph, plasma, synovial fluid, urine, and cerebrospinal fluid (22–28). Our group recently mapped the first peptidome transported by the human lymph. Over 300 self peptides were sequenced which derived from the catabolic processing of both intracellular and extracellular proteins (12). The peptidome comprised processed proteins derived from extracellular matrix proteins, cell adhesion molecules, and plasma membrane/receptors as well as an intracellular-derived peptidome consisting of fragments of cytosolic, nuclear (transcription factors and regulators of gene expression), mitochondrial, endosomal, Golgi, and endoplasmic reticulum proteins (12).

Peptide quantification by ^14^N/^15^N labeling and amino acid sequencing from 2D-DIGE spots indicated that many peptides were present in human lymph in at least nanomolar concentrations (12) and analysis of peptide half life in biological fluids indicated a stability of over 24 h (22).

Collectively all the experimental findings point to the lymph as an important biological fluid that transport the tissue “omics” (proteomes, degradomes, peptidomes) to the draining lymph nodes to convey a snapshot of each parenchymal organ in physiological and pathological conditions.

## Processing the Lymph and Plasma Degradome and Peptidome

Two major advances have improved our capability in identifying the lymph and plasma degradome and peptidome’s processing pathways; (i) improved mass spectrometric techniques, which allow high confidence peptide identification and correct amino acid assignment and (ii) increased representation of proteins in databases (Brenda, CutD, MEROPS), which facilitated prediction of the processing enzymes involved in peptide cleavage. Analysis of the human lymph- and plasma-carried peptidome identified peptides derived from both intra and extracellular sources and mapped several proteases likely involved in peptide processing including caspases, cathepsins, MMPs, plasmin, kallikreins, calpains, and granzymes (4, 21, 22, 24, 29–31). Peptides derived from intracellular proteins are likely released by damaged and apoptotic cells, several of which are normally found in the lymph (17, 32). These proteins could be cleaved by the proteasome, furins, calpains, cytosolic proteases, and caspases as well as extracellular proteases. Several peptides cleaved by cathepsins and other endosomal proteases were also found. These peptides were likely released from endosomal compartments during exosome exocytosis or processed extracellularly by released cathepsins (33). Peptides generated by the processing of matrix proteins and collagens could be generated by MMPs and ADAMs whose activity controls the constant remodeling of the extracellular matrix to accommodate organ growth, cell migration, and cell replenishment (34–36). Surface receptors, adhesion molecules, growth factors, and cytokines/chemokine, represent another category of processed peptides found in lymphatic fluid (37, 38). Additionally, products deriving from the complement cascade, thrombin and plasmin peptidases, and the kallikrein system were also found in the lymph.

Several other studies mapped the peptidome present in plasma, serum, and other biological fluids in physiological and pathological conditions including various types of cancer, inflammatory, and degenerative pathologies (4, 21, 22, 24, 29–31, 39, 40). Altogether, two major conclusions can be derived from these analyses:

(i) there is a great variety of processing pathways involved in the formation of the degradome/peptidome present in different biological fluids and
(ii) the degradome/peptidome profile changes according to the physiological or pathological state of the organ from where the lymph/plasma are collected.

Indeed, in pathological conditions, the mapped peptidome/degradome is highly enriched with new peptides as compared to the peptidome/degradome found in healthy physiological conditions (4, 7, 21, 22, 24, 29, 31, 39, 41, 42). This reflects the increased number of peptide fragments cleaved by proteases up-regulated during inflammation (21, 22, 24).

## MHC II Loading of the Self Peptidome

MHC II peptide complexes can be loaded in late endosomal MIIC compartments, early endosomes and at the plasma membrane (43–53). In the late endosomal compartment, antigen processing is dependent on cysteine, aspartic and asparagine endo-peptidases, and MHC II loading depends on the editing molecule HLA-DM (54–57). In early endosomes the antigen processing and MHC II loading is cathepsins and mostly HLA-DM-independent (49, 53, 58–60). Similarly extracellular peptides can be loaded at the cell surface either on empty MHC II molecules or through peptide exchange (47, 50, 52, 61–63).

Having distinct MHC class II loading compartments allows presentation of a larger array of peptides. Indeed many of the peptides loaded in recycling/early endosomes and at the cell surface are low affinity and are eliminated by HLA-DM in endosomal compartments (64). Thus, if the MIIC endosomal antigen processing and loading machinery restrict the array of presented peptides by kinetic stability and HLA-DM editing, generating an overall higher affinity, higher stability, long-lived MHC II peptidome (65), the HLA-DM-independent pathway generates a broader, lower stability/easily exchangeable MHC II peptidome.

These two different pathways are exploited by the antigen-presenting cells (APC) to control immunogenicity (65, 66).

Indeed the early endosomes/plasma membrane MHC II loading pathway is active in immature dendritic cells (DC) and down-regulated upon DC maturation (50–52, 64). As a result immature DC present an overall broader MHC II peptidome that includes low affinity/stability peptides which, by diluting the high affinity self peptides, contribute to the maintenance of self tolerance. The importance of maintaining a broader MHC II peptidome under physiological conditions is further supported by the notion that in immature DC and non-stimulated B cells the HLA-DM editing activity is down regulated, within the MHC II endosomal pathway, by HLA-DO; resulting in decreased presentation of high affinity self peptides that could induce autoimmunity (66, 67). Upon APC maturation/activation surface MHC II loading is shut off (50–52) and HLA-DM editing activity is up-regulated (66, 67). This would favor presentation of high affinity pathogen-derived peptides to generate immunity.

## MHC II Presentation of the Self Peptidome

Different mechanisms ensure that tissue-derived self antigens are constantly presented to the immune system for the maintenance of central and peripheral self tolerance (68–70).

In the thymus, medullary epithelial cells (mTEC), conventional dendritic cells (cDC) (Sirpα^+^ CD11c^+^ CD8^−^), CD8^+^ DC (Sirpα^−^ CD11c^+^ CD8^+^), and plasmacytoid DC delete immature thymocytes with high affinity for the self MHC II peptidome (71). These populations of APC display an MHC II-bound peptidome derived from exogenous antigens, acquired through phagocytosis, and endogenous antigen acquired through autophagy (69, 72). Additionally, a subset of mTEC expresses the transcriptional regulator AIRE which promotes expression of tissue-specific antigens, expanding the antigen repertoire to be presented (73). Since mTEC are equipped with all the proteins associated with the antigen processing and presentation machinery, they can directly process the AIRE-expressed antigens. Indeed the presence of autophagosomes in these cells indicates that antigens could enter the endosomal tract through autophagy (72). On the other hand several reports also indicate that mTEC can hand over AIRE-acquired antigens to cDC for thymic selection (74).

Self-antigens in the thymus can also give rise to natural or thymic T regs, through an avidity-dependent selection process. The APC controlling the formation of Treg are the same as those involved in the process of negative selection (75).

Cells that escape thymic deletion are tolerized in the periphery through anergy or Treg-mediated suppression by tissue and nodal resident dendritic cells (DCs) and macrophages (MΦ) which continuously process and present the self proteome of parenchymal organs. Additionally, AIRE-independent mechanisms have also been described in the periphery which mediate expression of tissue-specific self antigens by lymphatic endothelial cells, further expanding the presentation of the self proteome (76).

All the above described mechanisms depend on self-antigen delivery to endosomal compartments, through endocytosis or autophagy. Thus, they generate an MHCII peptidome mostly restricted by endosomal processing enzymes (77).

Recently, circulating cDC have been shown to promote both central and peripheral tolerance by displaying circulating self-antigens to immature thymocytes or mature peripheral T cells (78, 79). Indeed, even though it has been known for some time that intrathymic injection of organ specific APC induce long lasting tolerance to the organ self antigens (80, 81) it was the Goldschneider group that linked thymic tolerance, to extrathymic self antigens, to the role of cDC (82, 83). Work from his group and others indicated that under physiological conditions migratory DC transport self-antigens to mediate thymic negative selection or peripheral T cell anergy and Treg differentiation. In both humans and mice, circulating DC differentiate in a FLT3-dependent manner and are CD11c^+^, CCR7^+^, CD103^+^, and express high levels of MHC II and intermediate expression of co-stimulatory molecules (84). Importantly, migratory DC do not only rely on endosomal processing to display the MHC II peptidome but are capable of loading exogenous peptides as well. Indeed peptides have been shown to induce thymic negative selection not only when directly injected in the thymus (85) but also when injected in the blood stream (78, 86) or in the peritoneum (87–90) which is connected to mesenteric lymphatic drainage (91). Importantly, circulating self antigens have been shown to induce thymic negative selection at physiological concentrations, as the ones achieved on MHC II on the surface of APC (92).

## Conclusion

The interaction between MHC II-peptides and TCRs constitutes the molecular base for all CD4 T cell-mediated immune responses and the displayed MHC II peptidome is critical to the generation of tolerance, immunity, and autoimmunity. The loaded MHC II peptidome is selected based on MHC II affinity, presence or absence of HLA-DM activity, and arrays of available peptides. The degradome/peptidome present in the extracellular milieu and transported by the plasma, lymph, and other biological fluids could contributes to the generation of the MHC II peptidome. Indeed, in the last few years a series of proteomic analysis indicated that the amount of peptides present in biological fluids (lymph, blood, urine, peritoneal fluid) is much higher then what previously known, it is broader in repertoire and has a long half life (4, 21, 22, 24, 29–31, 39). These peptides could function in thymic negative selection similarly to the ones injected exogenously (78, 85–90, 92). Distinct from the peptidome generated in MIIC, the peptidome carried by the lymph/plasma is not restricted by endosomal proteases but originates from several other processing pathways, further expanding the self antigen repertoire presented by circulating DC for the maintenance of tolerance.

## Conflict of Interest Statement

The authors declare that the research was conducted in the absence of any commercial or financial relationships that could be construed as a potential conflict of interest.
